# Supplementary material for: One Health, One Hive: A scoping review of honey bees, climate change, pollutants, and antimicrobial resistance
Source: PLoS One. 2022 Feb 16;17(2):e0242393. doi: 10.1371/journal.pone.0242393 (PMC8849492; doi:10.1371/journal.pone.0242393)
Supplement: S1 Table — (PDF) [file pone.0242393.s002.pdf]

S3 Table. Complete search strings used in the searched databases.

Web of Science™ all databases[1]

1	TOPIC: (bee **OR** bees **OR** honey$bee* **OR** honeybee* **OR** honey **OR** beekeep* **OR** apiar* **OR** apicultur* **OR** “apis mellifera” **OR** apidae **OR** (hive **AND** (health **OR** success **OR** collapse **OR** product* **OR** stability)))
2	TOPIC: (((resistan* **OR** stewardship) **AND** (antibiotic* **OR** antimicrobial* **OR** anti-microbial* **OR** anti-bacterial* **OR** antibacterial* **OR** anti$viral* **OR** antiviral* **OR** anti$fungal* **OR** antifungal* **OR** anti$helminthic* **OR** antihelminthic* **OR** anthelmintic* **OR** anti$parasitic* **OR** antiparasitic* **OR** parasiticide* **OR** biocid* **OR** antiseptic* **OR** disinfectant* **OR** sterilant* **OR** sterili$er* **OR** chemosterilant* **OR** multidrug **OR** “multi$drug”)) **OR** AMR **OR** XDR **OR** TDR **OR** “super$bug*” **OR** superbug*)
3	TOPIC: ((climat* **NEAR** (chang* **OR** model$ing **OR** predict* **OR** resilience **OR** sensitivity)) **OR** (environment* **NEAR** chang*) **OR** “climate variability” **OR** “climatic variability” **OR** “global warm*” **OR** “greenhouse effect” **OR** “climate disaster” **OR** (storm **NOT** (electrical **OR** autonomic **OR** thyroid*)) **OR** wind **OR** “atmospheric pressure” **OR** season* **OR** precipitation **OR** snow* **OR** ice **OR** humid* **OR** rain* **OR** flood **OR** drought **OR** wildfire* **OR** (heat **NEAR** (wave* **OR** extreme* **OR** event)) **OR** temperature* **OR** cool **OR** cold **OR** weather **OR** “ultraviolet radiation” **OR** UV **OR** “El Nino-Southern Oscillation” **OR** “El Nino” **OR** “La Nina”)
4	TOPIC: (“air pollut*” **OR** “persistent organic pollut*” **OR** “particulate matter” **OR** “atmospheric contamin*” **OR** “atmospheric pollut*” **OR** “volatile organic compound*” **OR** “volatile organic pollutant” **OR** VOC **OR** VOCS **OR** “ambient air pollution” **OR** “household air pollution” **OR** “criteria air pollutant*” **OR** “biological air pollutant*” **OR** “physical pollutant*” **OR** “chemical pollutant*” **OR** gases **OR** ((“fossil fuel” **OR** industr*) **AND** pollut*) **OR** ((air **OR** water* **OR** soil) **AND** (contamin* **OR** toxic* **OR** “environment* health” **OR** quality **OR** disease* **OR** particulate* **OR** metal **OR** metals **OR** lead **OR** lead$II* **OR** Pb **OR** pb$+ **OR** zinc* **OR** Zn **OR** Zn$+ **OR** silver* **OR** Ag **OR** Ag+ **OR** copper* **OR** Cu **OR** Cu$+ **OR** Gallium* **OR** Ga **OR** Ga$+ **OR** cobalt* **OR** Co **OR** Co$+ **OR** Mercury* **OR** Hg **OR** Hg$+ **OR** Arsenic* **OR** As **OR** As$+ **OR** Nickel* **OR** Ni **OR** Ni$+ **OR** vehicle* **OR** automobile* **OR** exhaust **OR** motorway* **OR** roadway* **OR** highway* **OR** freeway* **OR** road* **OR** traffic **OR** urban **OR** Nox **OR** “nitrogen oxides” **OR** ozone **OR** particle*)) **OR** dust **OR** dusts **OR** “PM 2$5” **OR** PM2$5 **OR** PM$10 **OR** “ultrafine particle*” **OR** “polycyclic aromatic hydrocarbon*” **OR** PAH **OR** POPS **OR** smog **OR** “water pollut*” **OR** (water* **AND** (potable **OR** healthy **OR** drink* **OR** safe **OR** suitab* **OR** palatable **OR** edible **OR** tap **OR** fresh **OR** supply **OR** “microbial contamina*”)) **OR** waterborne **OR** water$borne **OR** aquifer **OR** groundwater **OR** pesticid* **OR** herbicid* **OR** insecticid* **OR** acaricid* **OR** fungicid* **OR** molluscacid* **OR** larvicid* **OR** fumigant **OR** “anti$fouling agent*” **OR** “agricultural chemical*” **OR** agrochemical* **OR** (defoliant* **AND** (chemical* **OR** agent*)) **OR** (hazardous **AND** substance*) **OR** (toxic **AND** action*) **OR** “chemically$induced disorder*” **OR** furfural **OR** aculeximycin **OR** “aluminum phosphide” **OR** “chromated copper arsenate” **OR** CCA **OR** creosote)
5	#3 **OR** #4
6	#1 **AND** #2 **AND** #5

[1] The Web of Science database index manager includes the following databases: Web of Science Core Collection, BIOSIS Citation Index, BIOSIS Previews, CABI: CAB Abstracts®, Current Contents Connect, Data Citation Index, Derwent Innovations Index, FSTA® - the food science resource, KCI-Korean Journal Database, MEDLINE®, Russian Science Citation Index, SciELO Citation Index, and Zoological Record.

Scopus®

1	TITLE-ABS-KEY(bee **OR** bees **OR** honey?bee* **OR** honeybee* **OR** honey **OR** beekeep* **OR** apiar* **OR** apicultur* **OR** “apis mellifera” **OR** apidae **OR (**hive **AND (**health **OR** success **OR** collapse **OR** product* **OR** stability)))
2	TITLE-ABS-KEY(((resistan* **OR** stewardship) **AND** (antibiotic* **OR** antimicrobial* **OR** anti-microbial* **OR** anti-bacterial* **OR** antibacterial* **OR** anti?viral* **OR** antiviral* **OR** anti?fungal* **OR** antifungal* **OR** anti?helminthic* **OR** antihelminthic* **OR** anthelmintic* **OR** anti?parasitic* **OR** antiparasitic* **OR** parasiticide* **OR** biocid* **OR** antiseptic* **OR** disinfectant* **OR** sterilant* **OR** sterili?er* **OR** chemosterilant* **OR** multidrug **OR** “multi?drug”)) **OR** AMR **OR** XDR **OR** TDR **OR** “super?bug*” **OR** superbug*)
3	TITLE-ABS-KEY((climat* **w/15 (**chang* **OR** model?ing **OR** predict* **OR** resilience **OR** sensitivity)) **OR** (environment* **w/15** chang*) **OR** “climate variability” **OR** “climatic variability” **OR “**global warm*” **OR** “greenhouse effect” **OR** “climate disaster” **OR** (storm **AND NOT** (electrical **OR** autonomic **OR** thyroid*)) **OR** wind **OR** “atmospheric pressure” **OR** season* **OR** precipitation **OR** snow* **OR** ice **OR** humid* **OR** rain* **OR** flood **OR** drought **OR** wildfire* **OR (**heat **w/15** (wave* **OR** extreme* **OR** event)) **OR** temperature* **OR** cool **OR** cold **OR** weather **OR** “ultraviolet radiation” **OR** UV **OR** “El Nino-Southern Oscillation” **OR** “El Nino” **OR** “La Nina”)
4	TITLE-ABS-KEY(“air pollut*” **OR** “persistent organic pollut*” **OR** “particulate matter” **OR** “atmospheric contamin*” **OR** “atmospheric pollut*” **OR** “volatile organic compound*” **OR** “volatile organic pollutant” **OR** VOC **OR** VOCS **OR** “ambient air pollution” **OR** “household air pollution” **OR** “criteria air pollutant*” **OR** “biological air pollutant*” **OR** “physical pollutant*” **OR** “chemical pollutant*” **OR** gases **OR** ((“fossil fuel” **OR** industr*) **AND** pollut*) **OR** ((air **OR** water* **OR** soil) **AND** (contamin* **OR** toxic* **OR** “environment* health” **OR** quality **OR** disease* **OR** particulate* **OR** metal **OR** metals **OR** lead **OR** lead?II* **OR** Pb **OR** pb?+ **OR** zinc* **OR** Zn **OR** Zn?+ **OR** silver* **OR** Ag **OR** Ag+ **OR** copper* **OR** Cu **OR** Cu?+ **OR** Gallium* **OR** Ga **OR** Ga?+ **OR** cobalt* **OR** Co **OR** Co?+ **OR** Mercury* **OR** Hg **OR** Hg?+ **OR** Arsenic* **OR** As **OR** As?+ **OR** Nickel* **OR** Ni **OR** Ni?+ **OR** vehicle* **OR** automobile* **OR** exhaust **OR** motorway* **OR** roadway* **OR** highway* **OR** freeway* **OR** road* **OR** traffic **OR** urban **OR** Nox **OR** “nitrogen oxides” **OR** ozone **OR** particle*)) **OR** dust **OR** dusts **OR** PM?2?5 **OR** PM?10 **OR** “ultrafine particle*” **OR** “polycyclic aromatic hydrocarbon*” **OR** PAH **OR** POPS **OR** smog **OR** “water pollut*” **OR** (water* **AND** (potable **OR** healthy **OR** drink* **OR** safe **OR** suitab* **OR** palatable **OR** edible **OR** tap **OR** fresh **OR** supply **OR** “microbial contamina*”)) **OR** waterborne **OR** water?borne **OR** aquifer **OR** groundwater **OR** pesticid* **OR** herbicid* **OR** insecticid* **OR** acaricid* **OR** fungicid* **OR** molluscacid* **OR** larvicid* **OR** fumigant **OR** “anti?fouling agent*” **OR** “agricultural chemical*” **OR** agrochemical* **OR** (defoliant* **AND** (chemical* **OR** agent*)) **OR** (hazardous **AND** substance*) **OR** (toxic **AND** action*) **OR** “chemically?induced disorder*” **OR** furfural

	**OR** aculeximycin **OR** “aluminum phosphide” **OR** “chromated copper arsenate” **OR** CCA **OR** creosote)
5	#3 **OR** #4
6	#1 **AND** #2 **AND** #5

Embase® via Ovid®

1	(bee **OR** bees **OR** honey?bee* **OR** honeybee* **OR** honey **OR** beekeep* **OR** apiar* **OR** apicultur* **OR** apis mellifera **OR** apidae **OR (**hive **AND** (health **OR** success **OR** collapse **OR** product* **OR** stability))).mp. [mp=title, abstract, heading word, drug trade name, original title, device manufacturer, drug manufacturer, device trade name, keyword, floating subheading word, candidate term word]
2	(((resistan* **OR** stewardship) **AND** (antibiotic* **OR** antimicrobial* **OR** anti-microbial* **OR** anti-bacterial* **OR** antibacterial* **OR** anti?viral* **OR** antiviral* **OR** anti?fungal* **OR** antifungal* **OR** anti?helminthic* **OR** antihelminthic* **OR** anthelmintic* **OR** anti?parasitic* **OR** antiparasitic* **OR** parasiticide* **OR** biocid* **OR** antiseptic* **OR** disinfectant* **OR** sterilant* **OR** sterili?er* **OR** chemosterilant* **OR** multidrug **OR** multi?drug)) **OR** AMR **OR** XDR **OR** TDR **OR** super?bug* **OR** superbug*).mp. [mp=title, abstract, heading word, drug trade name, original title, device manufacturer, drug manufacturer, device trade name, keyword, floating subheading word, candidate term word]
3	((climat* **adj15** (chang* **OR** model?ing **OR** predict* **OR** resilience **OR** sensitivity)) **OR** (environment* **adj15** chang*) **OR** climate variability **OR** climatic variability **OR** global warm* **OR** greenhouse effect **OR** climate disaster **OR** (storm **NOT** (electrical **OR** autonomic **OR** thyroid*)) **OR** wind **OR** atmospheric pressure **OR** season* **OR** precipitation **OR** snow* **OR** ice **OR** humid* **OR** rain* **OR** flood **OR** drought **OR** wildfire* **OR** (heat **adj15** (wave* **OR** extreme* **OR** event)) **OR** temperature* **OR** cool **OR** cold **OR** weather **OR** ultraviolet radiation **OR** UV **OR** El Nino-Southern Oscillation **OR** El Nino **OR** La Nina).mp. [mp=title, abstract, heading word, drug trade name, original title, device manufacturer, drug manufacturer, device trade name, keyword, floating subheading word, candidate term word]
4	(air pollut* **OR** persistent organic pollut* **OR** particulate matter **OR** atmospheric contamin* **OR** atmospheric pollut* **OR** volatile organic compound* **OR** volatile organic pollutant **OR** VOC **OR** VOCS **OR** ambient air pollution **OR** household air pollution **OR** criteria air pollutant* **OR** biological air pollutant* **OR** physical pollutant* **OR** chemical pollutant* **OR** gases **OR** ((fossil fuel **OR** industr*) **AND** pollut*) **OR** ((air **OR** water* **OR** soil) **AND** (contamin* **OR** toxic* **OR** environment* health **OR** quality **OR** disease* **OR** particulate* **OR** metal **OR** metals **OR** lead **OR** lead?II* **OR** Pb **OR** pb?+ **OR** zinc* **OR** Zn **OR** Zn?+ **OR** silver* **OR** Ag **OR** Ag+ **OR** copper* **OR** Cu **OR** Cu?+ **OR** Gallium* **OR** Ga **OR** Ga?+ **OR** cobalt* **OR** Co **OR** Co?+ **OR** Mercury* **OR** Hg **OR** Hg?+ **OR** Arsenic* **OR** As **OR** As?+ **OR** Nickel* **OR** Ni **OR** Ni?+ **OR** vehicle* **OR** automobile* **OR** exhaust **OR** motorway* **OR** roadway* **OR** highway* **OR** freeway* **OR** road* **OR** traffic **OR** urban **OR** Nox **OR** nitrogen oxides **OR** ozone **OR** particle*)) **OR** dust **OR** dusts **OR** PM?2?5 **OR** PM?10 **OR** ultrafine particle* **OR** polycyclic aromatic hydrocarbon* **OR** PAH **OR** POPS **OR** smog **OR** water pollut* **OR** (water* **AND** (potable **OR** healthy **OR** drink* **OR** safe **OR** suitab* **OR** palatable **OR** edible **OR** tap **OR** fresh **OR** supply **OR** microbial contamina*)) **OR** waterborne **OR** water?borne **OR** aquifer **OR**

	groundwater **OR** pesticid* **OR** herbicid* **OR** insecticid* **OR** acaricid* **OR** fungicid* **OR** molluscacid* **OR** larvicid* **OR** fumigant **OR** anti?fouling agent* **OR** agricultural chemical* **OR** agrochemical* **OR** (defoliant* **AND** (chemical* **OR** agent*)) **OR** (hazardous **AND** substance*) **OR** (toxic **AND** action*) **OR** chemically?induced disorder* **OR** furfural **OR** aculeximycin **OR** aluminum phosphide **OR** chromated copper arsenate **OR** CCA **OR** creosote).mp. [mp=title, abstract, heading word, drug trade name, original title, device manufacturer, drug manufacturer, device trade name, keyword, floating subheading word, candidate term word]
5	3 **OR** 4
6	1 **AND** 2 **AND** 5

AGRICOLA™ via ProQuest®

1	noft(bee **OR** bees **OR** honey*bee* **OR** honeybee* **OR** honey **OR** beekeep* **OR** apiar* **OR** apicultur* **OR** **"**apis mellifera" **OR** apidae **OR** **(**hive **AND** **(**health **OR** success **OR** collapse **OR** product* **OR** stability)))
2	noft(((resistan* **OR** stewardship) **AND** (antibiotic* **OR** antimicrobial* **OR** anti-microbial* **OR** anti-bacterial* **OR** antibacterial* **OR** anti*viral* **OR** antiviral* **OR** anti*fungal* **OR** antifungal* **OR** anti*helminthic* **OR** antihelminthic* **OR** anthelmintic* **OR** anti*parasitic* **OR** antiparasitic* **OR** parasiticide* **OR** biocid* **OR** antiseptic* **OR** disinfectant* **OR** sterilant* **OR** sterili*er* **OR** chemosterilant* **OR** multidrug **OR** **"**multi*drug")) **OR** AMR **OR** XDR **OR** TDR **OR** "super*bug*" **OR** superbug*)
3	noft((climat* **NEAR/15** **(**chang* **OR** model*ing **OR** predict* **OR** resilience **OR** sensitivity)) **OR** (environment* **NEAR/15** chang*) **OR** "climate variability" **OR** "climatic variability" **OR** **"**global warm*" **OR** "greenhouse effect" **OR** "climate disaster" **OR** (storm **NOT** (electrical **OR** autonomic **OR** thyroid*)) **OR** wind **OR** "atmospheric pressure" **OR** season* **OR** precipitation **OR** snow* **OR** ice **OR** humid* **OR** rain* **OR** flood **OR** drought **OR** wildfire* **OR** (heat **NEAR/15** (wave* **OR** extreme* **OR** event)) **OR** temperature* **OR** cool **OR** cold **OR** weather **OR** "ultraviolet radiation" **OR** UV **OR** "El Nino-Southern Oscillation" **OR** "El Nino" **OR** "La Nina")
4	noft("air pollut*" **OR** "persistent organic pollut*" **OR** "particulate matter" **OR** "atmospheric contamin*" **OR** "atmospheric pollut*" **OR** "volatile organic compound*" **OR** "volatile organic pollutant" **OR** VOC **OR** VOCS **OR** "ambient air pollution" **OR** "household air pollution" **OR** "criteria air pollutant*" **OR** "biological air pollutant*" **OR** "physical pollutant*" **OR** "chemical pollutant*" **OR** gases **OR** (("fossil fuel" **OR** industr*) **AND** pollut*) **OR** ((air **OR** water* **OR** soil) **AND** (contamin* **OR** toxic* **OR** "environment* health" **OR** quality **OR** disease* **OR** particulate* **OR** metal **OR** metals **OR** lead **OR** lead*II* **OR** Pb **OR** pb*+ **OR** zinc* **OR** Zn **OR** Zn*+ **OR** silver* **OR** Ag **OR** Ag+ **OR** copper* **OR** Cu **OR** Cu*+ **OR** Gallium* **OR** Ga **OR** Ga*+ **OR** cobalt* **OR** Co **OR** Co*+ **OR** Mercury* **OR** Hg **OR** Hg*+ **OR** Arsenic* **OR** As **OR** As*+ **OR** Nickel* **OR** Ni **OR** Ni*+ **OR** vehicle* **OR** automobile* **OR** exhaust **OR** motorway* **OR** roadway* **OR** highway* **OR** freeway* **OR** road* **OR** traffic **OR** urban **OR** Nox **OR** "nitrogen oxides" **OR** ozone **OR** particle*)) **OR** dust **OR** dusts **OR** PM*2*5 **OR** PM*10 **OR** "ultrafine particle*" **OR** "polycyclic aromatic hydrocarbon*" **OR** PAH **OR** POPS **OR** smog **OR** "water pollut*" **OR** (water* **AND** (potable **OR** healthy **OR** drink* **OR** safe **OR** suitab* **OR** palatable **OR** edible **OR** tap **OR** fresh **OR** supply **OR** "microbial contamina*")) **OR** waterborne **OR** water*borne **OR** aquifer **OR** groundwater **OR**

	pesticid* **OR** herbicid* **OR** insecticid* **OR** acaricid* **OR** fungicid* **OR** molluscacid* **OR** larvicid* **OR** fumigant **OR** “anti*fouling agent*” **OR** “agricultural chemical*” **OR** agrochemical* **OR** (defoliant* **AND** (chemical* **OR** agent*)) **OR** (hazardous **AND** substance*) **OR** (toxic **AND** action*) **OR** “chemically*induced disorder*” **OR** furfural **OR** aculeximycin **OR** “aluminum phosphide” **OR** “chromated copper arsenate” **OR** CCA **OR** creosote)
5	3 **OR** 4
6	1 **AND** 2 **AND** 5
