# Supplementary material for: One Health, One Hive: A scoping review of honey bees, climate change, pollutants, and antimicrobial resistance
Source: PLoS One. 2022 Feb 16;17(2):e0242393. doi: 10.1371/journal.pone.0242393 (PMC8849492; doi:10.1371/journal.pone.0242393)
Supplement: S1 File — (PDF) [file pone.0242393.s004.pdf]

Not that kind of superbug: A scoping review of AMR and honey bees in a changing environment

Objective

The objective of this scoping review is to examine the range, extent, and nature of published literature on the relationship between antimicrobial resistance (AMR) and honey bees in the context of a changing planet. Specifically, the effects of climate change and pollutants on AMR in honey bees will be investigated.

Research Question

Primary research question

How does climate change and/or pollutants affect antimicrobial resistance in honey bees?

Methods

The scoping review will follow the framework outlined in the Joanna Briggs Institute Reviewer's Manual which includes identifying the research question, finding relevant studies, selecting studies, charting the data, and reporting the results (1-5). Reporting guidelines set by Tricco et al. in their scoping review extension of the PRISMA checklist will be followed (6,7).

1.1 Search Methods

- Electronic Database Search — a comprehensive search string will be used to search the following databases:
  - o Web of Science™[1] covers multi-disciplinary literature in the fields of nature, health, and social sciences by sourcing multiple databases such as MEDLINE® and CAB Abstracts®;
  - o Scopus® provides literature related to medicine, technology, science, and social sciences;
  - o Embase® via Ovid® covers a vast range of biomedical sciences journals, with an added focus on European studies not present in MEDLINE®; and
  - o AGRICOLA™ via ProQuest® sources from the United States National Agricultural Library to retrieve global literature on the topic of agriculture.
- Snowball Search — a snowball search will be conducted on the reference lists of all the literature that meet the inclusion criteria to capture any articles missed by the initial electronic database search.
- Hand Search — specific journals relevant to our focus topics will be manually searched for any articles not captured by the database search. The "analyze results" feature in the Web of Science™ indexing service was used to determine which journals to hand search. This feature allowed us to see which journals contained the

[1] The Web of Science database index manager includes the following databases: Web of Science Core Collection, BIOSIS Citation Index, BIOSIS Previews, CABI: CAB Abstracts®, Current Contents Connect, Data Citation Index, Derwent Innovations Index, FSTA® - the food science resource, KCI-Korean Journal Database, MEDLINE®, Russian Science Citation Index, SciELO Citation Index, Zoological Record.

most relevant topics by inputting our search strings and analyzing which journals had a large proportion of hits. Our chosen journals are presented below:

- *Environmental Pollution*: journal covering a wide range of topics on environmental pollution with a focus on those related to human and ecosystem health.
- *Climatic Change*: journal covering cross-disciplinary literature related to climate change.
- *Antimicrobial agents and chemotherapy®*: journal covering research on the foundational mechanisms and therapeutic applications of antimicrobial drugs.
- *Journal of Apicultural Research:* peer-reviewed journal covering research and literature on the topic of bees.

1.2 Search String

Component	Search Terms
Honey Bees	(bee **OR** bees **OR** honey?bee* **OR** honeybee* **OR** honey **OR** beekeep* **OR** apiar* **OR** apicultur* **OR** **"**apis mellifera" **OR** apidae **OR** **(**hive **AND** **(**health **OR** success **OR** collapse **OR** product* **OR** stability)))<br><br>AND
Antimicrobial Resistance (AMR)	(((resistan* **OR** stewardship) **AND** (antibiotic* **OR** antimicrobial* **OR** anti-microbial* **OR** anti-bacterial* **OR** antibacterial* **OR** anti?viral* **OR** antiviral* **OR** anti?fungal* **OR** antifungal* **OR** anti?helminthic* **OR** antihelminthic* **OR** anthelmintic* **OR** anti?parasitic* **OR** antiparasitic* **OR** parasiticide* **OR** biocid* **OR** antiseptic* **OR** disinfectant* **OR** sterilant* **OR** sterili?er* **OR** chemosterilant* **OR** multidrug **OR** **"**multi?drug")) **OR** AMR **OR** XDR **OR** TDR **OR** "super?bug*" **OR** superbug*)<br><br>AND
Climate Change	((climat* **NEAR** **(**chang* **OR** model?ing **OR** predict* **OR** resilience **OR** sensitivity)) **OR** (environment* **NEAR** chang*) **OR** "climate variability" **OR** "climatic variability" **OR** **"**global warm*" **OR** "greenhouse effect" **OR** "climate disaster" **OR** (storm **NOT** (electrical **OR** autonomic **OR** thyroid*)) **OR** wind **OR** "atmospheric pressure" **OR** season* **OR** precipitation **OR** snow* **OR** ice **OR** humid* **OR** rain* **OR** flood **OR** drought **OR** wildfire* **OR** (heat **NEAR** (wave* **OR** extreme* **OR** event)) **OR** temperature* **OR** cool **OR** cold **OR** weather **OR** "ultraviolet

	radiation” **OR** UV **OR** “El Nino-Southern Oscillation” **OR** “El Nino” **OR** “La Nina”)<br><br>OR
Environmental pollutants	(“air pollut*” **OR** “persistent organic pollut*” **OR** “particulate matter” **OR** “atmospheric contamin*” **OR** “atmospheric pollut*” **OR** “volatile organic compound*” **OR** “volatile organic pollutant” **OR** VOC **OR** VOCS **OR** “ambient air pollution” **OR** “household air pollution” **OR** “criteria air pollutant*” **OR** “biological air pollutant*” **OR** “physical pollutant*” **OR** “chemical pollutant*” **OR** gases **OR** **((**“fossil fuel” **OR** industr*) **AND** pollut*) **OR** ((air **OR** water* **OR** soil) **AND** (contamin* **OR** toxic* **OR** “environment* health” **OR** quality **OR** disease* **OR** particulate* **OR** metal **OR** metals **OR** lead **OR** lead?II* **OR** Pb **OR** pb?+ **OR** zinc* **OR** Zn **OR** Zn?+ **OR** silver* **OR** Ag **OR** Ag+ **OR** copper* **OR** Cu **OR** Cu?+ **OR** Gallium* **OR** Ga **OR** Ga?+ **OR** cobalt* **OR** Co **OR** Co?+ **OR** Mercury* **OR** Hg **OR** Hg?+ **OR** Arsenic* **OR** As **OR** As?+ **OR** Nickel* **OR** Ni **OR** Ni?+ **OR** vehicle* **OR** automobile* **OR** exhaust **OR** motorway* **OR** roadway* **OR** highway* **OR** freeway* **OR** road* **OR** traffic **OR** urban **OR** Nox **OR** “nitrogen oxides” **OR** ozone **OR** particle*)) **OR** dust **OR** dusts **OR** PM?2?5 **OR** PM?10 **OR** “ultrafine particle*” **OR** “polycyclic aromatic hydrocarbon*” **OR** PAH **OR** POPS **OR** smog **OR** “water pollut*” **OR** (water* **AND** (potable **OR** healthy **OR** drink* **OR** safe **OR** suitab* **OR** palatable **OR** edible **OR** tap **OR** fresh **OR** supply **OR** “microbial contamina*”)) **OR** waterborne **OR** water?borne **OR** aquifer **OR** groundwater **OR** pesticid* **OR** herbicid* **OR** insecticid* **OR** acaricid* **OR** fungicid* **OR** molluscacid* **OR** larvicid* **OR** fumigant **OR** “anti?fouling agent*” **OR** “agricultural chemical*” **OR** agrochemical* **OR** (defoliant* **AND** (chemical* **OR** agent*)) **OR** (hazardous **AND** substance*) **OR** (toxic **AND** action*) **OR** “chemically?induced disorder*” **OR** furfural **OR** aculeximycin **OR** “aluminum phosphide” **OR** “chromated copper arsenate” **OR** CCA **OR** creosote)<br><br>OR

Specific combinations of the search strings used to retrieve our articles can be found in the Appendix

2. Screening

2.1 Restrictions

- No language restrictions will be placed on the search string.
- No date restrictions will be placed on the search string.

2.2 Level 1 Screening

- A stacked questionnaire will be used to screen the titles and abstracts captured by the initial searches by two independent reviewers.
- If the article fully or partially meets the inclusion criteria (i.e. all screening questions are answered either 'yes' or 'unsure'), the article will proceed to Level 2 screening.
- If the article meets any exclusion criteria, it will not proceed to Level 2 screening.
- A second independent reviewer will confirm exclusion of the article.
- Google Translate™ will be used to aid screening non-English titles and abstracts.

2.3 Level 2 Screening

- Two independent reviewers will review the full article based on the inclusion and exclusion criteria.
- In Level 2 screening, only articles that meet all the inclusion criteria will be included in the review. 'Unsure' is not an option.

2.4 Inclusion Criteria

- There is no language restriction at Level 1 Screening; all published literature will be examined.
- There is no date restriction; published literature from all available dates will be examined.
- Describes research on the relationship between AMR and/or honey bees in the context of climatic variables and environmental pollutants:
    - Must include both AMR and honey bees as primary points of interest with at least partial inclusion of climatic and/or pollutant variables.
    - Antimicrobial resistance: the ability of a microbe (not limited to bacteria) to resist or reduce the effects of a drug or treatment meant to adversely affect its normal function. The primary focus is on drivers of AMR, hence novel drug testing will not be included.
    - Climate Change: change in climate that persists for a long period of time (decades or longer). Change in climate could be due to natural causes or anthropogenic (7). Climatic variables in the context of climate change will also be included under this limiter to capture relevant articles that do not directly correlate climatic variables to climate change.
    - Honey bees: organisms from the taxum *apis mellifera.*

2.5 Exclusion Criteria

- Books, book chapters, theses, dissertations

- Describes research that does not investigate AMR in honey bees.
- Describes research that does not focus on the impacts of climatic variables, and/or environmental pollutants on AMR in honey bees.
    - Research on AMR in honey bees but without any inclusion of climatic or pollution variables.
    - Research on the effects of climatic variables and pollutants on AMR but not related to the life or function of honey bees or hive health.

2.6 Screening Questions

Level 1: the following questions will be answered using a stacked form after screening each title and/or abstract:

- Does the research focus on beekeeping and/or honey bee hive health? (altering the normal function or behaviour of the hive or organism)
    - Yes
    - No
    - Unsure
- Does the research focus on AMR?
    - Yes
    - No
    - Unsure
- Does the research include climatic variables and/or environmental pollutants as points of interest?
    - Yes
    - No
    - Unsure
- Is it a journal article?
    - Yes
    - No
    - Unsure?

If 'unsure' or 'yes' is selected, confirm inclusions.

Level 2: the following questions will be answered after screening each full article:

- Does the research focus on beekeeping and/or honey bee hive health? (altering the normal function or behaviour of the hive or organism)
    - Yes
    - No
- Does the research focus on AMR? (not including in vitro or novel drug testing)
    - Yes
    - No
- Does the research mention the effects of climatic variables and/or environmental pollutants on AMR development?
    - Yes
    - No

2.7 Screening Process

Database	Date of search	Search Fields	# of Results
Web of Science™ all databases (Medline®, CAB®, etc..)	July 10th, 2019	No restrictions	1,099
Scopus®	July 10th, 2019	No restrictions	163
Embase® via Ovid®	July 10th, 2019	No restrictions	106
AGRICOLA™ via ProQuest®	July 10th, 2019	No restrictions	34

3. Data Collection and Analysis

3.1 Software

Mendeley© will be used for citation management for the scoping review. All eligible articles will be uploaded onto Mendeley©. The software will remove any duplicates.

Articles will then be uploaded onto DistillerSR® to undergo screening. Screening forms will be created using the software and will be used to determine eligibility.

Appendix

Web of Science™ all databases (Medline®, CAB®, etc..)

1	TOPIC: (bee **OR** bees **OR** honey$bee* **OR** honeybee* **OR** honey **OR** beekeep* **OR** apiar* **OR** apicultur* **OR** **"**apis mellifera" **OR** apidae **OR** **(**hive **AND** **(**health **OR** success **OR** collapse **OR** product* **OR** stability)))
2	TOPIC: (((resistan* **OR** stewardship) **AND** (antibiotic* **OR** antimicrobial* **OR** anti-microbial* **OR** anti-bacterial* **OR** antibacterial* **OR** anti$viral* **OR** antiviral* **OR** anti$fungal* **OR** antifungal* **OR** anti$helminthic* **OR** antihelminthic* **OR** anthelmintic* **OR** anti$parasitic* **OR** antiparasitic* **OR** parasiticide* **OR** biocid* **OR** antiseptic* **OR** disinfectant* **OR** sterilant* **OR** sterili$er* **OR** chemosterilant* **OR** multidrug **OR** **"**multi$drug")) **OR** AMR **OR** XDR **OR** TDR **OR** "super$bug*" **OR** superbug*)
3	TOPIC: ((climat* **NEAR** **(**chang* **OR** model$ing **OR** predict* **OR** resilience **OR** sensitivity)) **OR** (environment* **NEAR** chang*) **OR** "climate variability" **OR** "climatic variability" **OR** **"**global warm*" **OR** "greenhouse effect" **OR** "climate disaster" **OR** (storm **NOT** (electrical **OR** autonomic **OR** thyroid*)) **OR** wind **OR** "atmospheric pressure" **OR** season* **OR** precipitation **OR** snow* **OR** ice **OR** humid* **OR** rain* **OR** flood **OR** drought **OR** wildfire* **OR** **(**heat **NEAR** (wave* **OR** extreme* **OR** event)) **OR** temperature* **OR** cool **OR** cold **OR** weather **OR** "ultraviolet radiation" **OR** UV **OR** "El Nino-Southern Oscillation" **OR** "El Nino" **OR** "La Nina")

4	TOPIC: ("air pollut*" **OR** "persistent organic pollut*" **OR** "particulate matter" **OR** "atmospheric contamin*" **OR** "atmospheric pollut*" **OR** "volatile organic compound*" **OR** "volatile organic pollutant" **OR** VOC **OR** VOCS **OR** "ambient air pollution" **OR** "household air pollution" **OR** "criteria air pollutant*" **OR** "biological air pollutant*" **OR** "physical pollutant*" **OR** "chemical pollutant*" **OR** gases **OR** (("fossil fuel" **OR** industr*) **AND** pollut*) **OR** ((air **OR** water* **OR** soil) **AND** (contamin* **OR** toxic* **OR** "environment* health" **OR** quality **OR** disease* **OR** particulate* **OR** metal **OR** metals **OR** lead **OR** lead$II* **OR** Pb **OR** pb$+ **OR** zinc* **OR** Zn **OR** Zn$+ **OR** silver* **OR** Ag **OR** Ag+ **OR** copper* **OR** Cu **OR** Cu$+ **OR** Gallium* **OR** Ga **OR** Ga$+ **OR** cobalt* **OR** Co **OR** Co$+ **OR** Mercury* **OR** Hg **OR** Hg$+ **OR** Arsenic* **OR** As **OR** As$+ **OR** Nickel* **OR** Ni **OR** Ni$+ **OR** vehicle* **OR** automobile* **OR** exhaust **OR** motorway* **OR** roadway* **OR** highway* **OR** freeway* **OR** road* **OR** traffic **OR** urban **OR** Nox **OR** "nitrogen oxides" **OR** ozone **OR** particle*)) **OR** dust **OR** dusts **OR** "PM 2$5" **OR** PM2$5 **OR** PM$10 **OR** "ultrafine particle*" **OR** "polycyclic aromatic hydrocarbon*" **OR** PAH **OR** POPS **OR** smog **OR** "water pollut*" **OR** (water* **AND** (potable **OR** healthy **OR** drink* **OR** safe **OR** suitab* **OR** palatable **OR** edible **OR** tap **OR** fresh **OR** supply **OR** "microbial contamina*")) **OR** waterborne **OR** water$borne **OR** aquifer **OR** groundwater **OR** pesticid* **OR** herbicid* **OR** insecticid* **OR** acaricid* **OR** fungicid* **OR** molluscacid* **OR** larvicid* **OR** fumigant **OR** "anti$fouling agent*" **OR** "agricultural chemical*" **OR** agrochemical* **OR** (defoliant* **AND** (chemical* **OR** agent*)) **OR** (hazardous **AND** substance*) **OR** (toxic **AND** action*) **OR** "chemically$induced disorder*" **OR** furfural **OR** aculeximycin **OR** "aluminum phosphide" **OR** "chromated copper arsenate" **OR** CCA **OR** creosote)
5	#3 **OR** #4
6	#1 **AND** #2 **AND** #5

Scopus®

1	TITLE-ABS-KEY(bee **OR** bees **OR** honey?bee* **OR** honeybee* **OR** honey **OR** beekeep* **OR** apiar* **OR** apicultur* **OR** "apis mellifera" **OR** apidae **OR (**hive **AND (**health **OR** success **OR** collapse **OR** product* **OR** stability)))
2	TITLE-ABS-KEY(((resistan* **OR** stewardship) **AND** (antibiotic* **OR** antimicrobial* **OR** anti-microbial* **OR** anti-bacterial* **OR** antibacterial* **OR** anti?viral* **OR** antiviral* **OR** anti?fungal* **OR** antifungal* **OR** anti?helminthic* **OR** antihelminthic* **OR** anthelmintic* **OR** anti?parasitic* **OR** antiparasitic* **OR** parasiticide* **OR** biocid* **OR** antiseptic* **OR** disinfectant* **OR** sterilant* **OR** sterili?er* **OR** chemosterilant* **OR** multidrug **OR** "multi?drug")) **OR** AMR **OR** XDR **OR** TDR **OR** "super?bug*" **OR** superbug*)

3	TITLE-ABS-KEY((climat* **w/15 (**chang* **OR** model?ing **OR** predict* **OR** resilience **OR** sensitivity)) **OR** (environment* **w/15** chang*) **OR** “climate variability” **OR** “climatic variability” **OR “**global warm*” **OR** “greenhouse effect” **OR** “climate disaster” **OR** (storm **AND NOT** (electrical **OR** autonomic **OR** thyroid*)) **OR** wind **OR** “atmospheric pressure” **OR** season* **OR** precipitation **OR** snow* **OR** ice **OR** humid* **OR** rain* **OR** flood **OR** drought **OR** wildfire* **OR (**heat **w/15** (wave* **OR** extreme* **OR** event)) **OR** temperature* **OR** cool **OR** cold **OR** weather **OR** “ultraviolet radiation” **OR** UV **OR** “El Nino-Southern Oscillation” **OR** “El Nino” **OR** “La Nina”)
4	TITLE-ABS-KEY(“air pollut*” **OR** “persistent organic pollut*” **OR** “particulate matter” **OR** “atmospheric contamin*” **OR** “atmospheric pollut*” **OR** “volatile organic compound*” **OR** “volatile organic pollutant” **OR** VOC **OR** VOCS **OR** “ambient air pollution” **OR** “household air pollution” **OR** “criteria air pollutant*” **OR** “biological air pollutant*” **OR** “physical pollutant*” **OR** “chemical pollutant*” **OR** gases **OR** ((“fossil fuel” **OR** industr*) **AND** pollut*) **OR** ((air **OR** water* **OR** soil) **AND** (contamin* **OR** toxic* **OR** “environment* health” **OR** quality **OR** disease* **OR** particulate* **OR** metal **OR** metals **OR** lead **OR** lead?II* **OR** Pb **OR** pb?+ **OR** zinc* **OR** Zn **OR** Zn?+ **OR** silver* **OR** Ag **OR** Ag+ **OR** copper* **OR** Cu **OR** Cu?+ **OR** Gallium* **OR** Ga **OR** Ga?+ **OR** cobalt* **OR** Co **OR** Co?+ **OR** Mercury* **OR** Hg **OR** Hg?+ **OR** Arsenic* **OR** As **OR** As?+ **OR** Nickel* **OR** Ni **OR** Ni?+ **OR** vehicle* **OR** automobile* **OR** exhaust **OR** motorway* **OR** roadway* **OR** highway* **OR** freeway* **OR** road* **OR** traffic **OR** urban **OR** Nox **OR** “nitrogen oxides” **OR** ozone **OR** particle*)) **OR** dust **OR** dusts **OR** PM?2?5 **OR** PM?10 **OR** “ultrafine particle*” **OR** “polycyclic aromatic hydrocarbon*” **OR** PAH **OR** POPS **OR** smog **OR** “water pollut*” **OR** (water* **AND** (potable **OR** healthy **OR** drink* **OR** safe **OR** suitab* **OR** palatable **OR** edible **OR** tap **OR** fresh **OR** supply **OR** “microbial contamina*”)) **OR** waterborne **OR** water?borne **OR** aquifer **OR** groundwater **OR** pesticid* **OR** herbicid* **OR** insecticid* **OR** acaricid* **OR** fungicid* **OR** molluscacid* **OR** larvicid* **OR** fumigant **OR** “anti?fouling agent*” **OR** “agricultural chemical*” **OR** agrochemical* **OR** (defoliant* **AND** (chemical* **OR** agent*)) **OR** (hazardous **AND** substance*) **OR** (toxic **AND** action*) **OR** “chemically?induced disorder*” **OR** furfural **OR** aculeximycin **OR** “aluminum phosphide” **OR** “chromated copper arsenate” **OR** CCA **OR** creosote)
5	#3 **OR** #4
6	#1 **AND** #2 **AND** #5

Embase® via Ovid®

1	(bee **OR** bees **OR** honey?bee* **OR** honeybee* **OR** honey **OR** beekeep* **OR** apiar* **OR** apicultur* **OR** apis mellifera **OR** apidae **OR (**hive **AND** (health **OR** success

	**OR** collapse **OR** product* **OR** stability))).mp. [mp=title, abstract, heading word, drug trade name, original title, device manufacturer, drug manufacturer, device trade name, keyword, floating subheading word, candidate term word]
2	(((resistan* **OR** stewardship) **AND** (antibiotic* **OR** antimicrobial* **OR** anti-microbial* **OR** anti-bacterial* **OR** antibacterial* **OR** anti?viral* **OR** antiviral* **OR** anti?fungal* **OR** antifungal* **OR** anti?helminthic* **OR** antihelminthic* **OR** anthelmintic* **OR** anti?parasitic* **OR** antiparasitic* **OR** parasiticide* **OR** biocid* **OR** antiseptic* **OR** disinfectant* **OR** sterilant* **OR** sterili?er* **OR** chemosterilant* **OR** multidrug **OR** multi?drug)) **OR** AMR **OR** XDR **OR** TDR **OR** super?bug* **OR** superbug*).mp. [mp=title, abstract, heading word, drug trade name, original title, device manufacturer, drug manufacturer, device trade name, keyword, floating subheading word, candidate term word]
3	((climat* **adj15** (chang* **OR** model?ing **OR** predict* **OR** resilience **OR** sensitivity)) **OR** (environment* **adj15** chang*) **OR** climate variability **OR** climatic variability **OR** global warm* **OR** greenhouse effect **OR** climate disaster **OR** (storm **NOT** (electrical **OR** autonomic **OR** thyroid*)) **OR** wind **OR** atmospheric pressure **OR** season* **OR** precipitation **OR** snow* **OR** ice **OR** humid* **OR** rain* **OR** flood **OR** drought **OR** wildfire* **OR** (heat **adj15** (wave* **OR** extreme* **OR** event)) **OR** temperature* **OR** cool **OR** cold **OR** weather **OR** ultraviolet radiation **OR** UV **OR** El Nino-Southern Oscillation **OR** El Nino **OR** La Nina).mp. [mp=title, abstract, heading word, drug trade name, original title, device manufacturer, drug manufacturer, device trade name, keyword, floating subheading word, candidate term word]
4	(air pollut* **OR** persistent organic pollut* **OR** particulate matter **OR** atmospheric contamin* **OR** atmospheric pollut* **OR** volatile organic compound* **OR** volatile organic pollutant **OR** VOC **OR** VOCS **OR** ambient air pollution **OR** household air pollution **OR** criteria air pollutant* **OR** biological air pollutant* **OR** physical pollutant* **OR** chemical pollutant* **OR** gases **OR** ((fossil fuel **OR** industr*) **AND** pollut*) **OR** ((air **OR** water* **OR** soil) **AND** (contamin* **OR** toxic* **OR** environment* health **OR** quality **OR** disease* **OR** particulate* **OR** metal **OR** metals **OR** lead **OR** lead?II* **OR** Pb **OR** pb?+ **OR** zinc* **OR** Zn **OR** Zn?+ **OR** silver* **OR** Ag **OR** Ag+ **OR** copper* **OR** Cu **OR** Cu?+ **OR** Gallium* **OR** Ga **OR** Ga?+ **OR** cobalt* **OR** Co **OR** Co?+ **OR** Mercury* **OR** Hg **OR** Hg?+ **OR** Arsenic* **OR** As **OR** As?+ **OR** Nickel* **OR** Ni **OR** Ni?+ **OR** vehicle* **OR** automobile* **OR** exhaust **OR** motorway* **OR** roadway* **OR** highway* **OR** freeway* **OR** road* **OR** traffic **OR** urban **OR** Nox **OR** nitrogen oxides **OR** ozone **OR** particle*)) **OR** dust **OR** dusts **OR** PM?2?5 **OR** PM?10 **OR** ultrafine particle* **OR** polycyclic aromatic hydrocarbon* **OR** PAH **OR** POPS **OR** smog **OR** water pollut* **OR** (water* **AND** (potable **OR** healthy **OR** drink* **OR** safe **OR** suitab* **OR** palatable **OR** edible **OR** tap **OR** fresh **OR** supply **OR** microbial contamina*))

	**OR** waterborne **OR** water?borne **OR** aquifer **OR** groundwater **OR** pesticid* **OR** herbicid* **OR** insecticid* **OR** acaricid* **OR** fungicid* **OR** molluscacid* **OR** larvicid* **OR** fumigant **OR** anti?fouling agent* **OR** agricultural chemical* **OR** agrochemical* **OR** (defoliant* **AND** (chemical* **OR** agent*)) **OR** (hazardous **AND** substance*) **OR** (toxic **AND** action*) **OR** chemically?induced disorder* **OR** furfural **OR** aculeximycin **OR** aluminum phosphide **OR** chromated copper arsenate **OR** CCA **OR** creosote).mp. [mp=title, abstract, heading word, drug trade name, original title, device manufacturer, drug manufacturer, device trade name, keyword, floating subheading word, candidate term word]
5	3 **OR** 4
6	1 **AND** 2 **AND** 5

AGRICOLA™ via ProQuest®

1	noft(bee **OR** bees **OR** honey*bee* **OR** honeybee* **OR** honey **OR** beekeep* **OR** apiar* **OR** apicultur* **OR “**apis mellifera” **OR** apidae **OR (**hive **AND (**health **OR** success **OR** collapse **OR** product* **OR** stability)))
2	noft(((resistan* **OR** stewardship) **AND** (antibiotic* **OR** antimicrobial* **OR** anti-microbial* **OR** anti-bacterial* **OR** antibacterial* **OR** anti*viral* **OR** antiviral* **OR** anti*fungal* **OR** antifungal* **OR** anti*helminthic* **OR** antihelminthic* **OR** anthelmintic* **OR** anti*parasitic* **OR** antiparasitic* **OR** parasiticide* **OR** biocid* **OR** antiseptic* **OR** disinfectant* **OR** sterilant* **OR** sterili*er* **OR** chemosterilant* **OR** multidrug **OR “**multi*drug”)) **OR** AMR **OR** XDR **OR** TDR **OR** “super*bug*” **OR** superbug*)
3	noft((climat* **NEAR/15 (**chang* **OR** model*ing **OR** predict* **OR** resilience **OR** sensitivity)) **OR** (environment* **NEAR/15** chang*) **OR** “climate variability” **OR** “climatic variability” **OR “**global warm*” **OR** “greenhouse effect” **OR** “climate disaster” **OR** (storm **NOT** (electrical **OR** autonomic **OR** thyroid*)) **OR** wind **OR** “atmospheric pressure” **OR** season* **OR** precipitation **OR** snow* **OR** ice **OR** humid* **OR** rain* **OR** flood **OR** drought **OR** wildfire* **OR** (heat **NEAR/15** (wave* **OR** extreme* **OR** event)) **OR** temperature* **OR** cool **OR** cold **OR** weather **OR** “ultraviolet radiation” **OR** UV **OR** “El Nino-Southern Oscillation” **OR** “El Nino” **OR** “La Nina”)
4	noft(“air pollut*” **OR** “persistent organic pollut*” **OR** “particulate matter” **OR** “atmospheric contamin*” **OR** “atmospheric pollut*” **OR** “volatile organic compound*” **OR** “volatile organic pollutant” **OR** VOC **OR** VOCS **OR** “ambient air pollution” **OR** “household air pollution” **OR** “criteria air pollutant*” **OR** “biological air pollutant*” **OR** “physical pollutant*” **OR** “chemical pollutant*” **OR** gases **OR** ((“fossil fuel” **OR** industr*) **AND** pollut*) **OR** ((air **OR** water* **OR** soil) **AND** (contamin* **OR** toxic* **OR** “environment* health” **OR** quality **OR** disease* **OR** particulate* **OR** metal **OR** metals **OR** lead **OR** lead*II* **OR** Pb **OR**

	pb*+ **OR** zinc* **OR** Zn **OR** Zn*+ **OR** silver* **OR** Ag **OR** Ag+ **OR** copper* **OR** Cu **OR** Cu*+ **OR** Gallium* **OR** Ga **OR** Ga*+ **OR** cobalt* **OR** Co **OR** Co*+ **OR** Mercury* **OR** Hg **OR** Hg*+ **OR** Arsenic* **OR** As **OR** As*+ **OR** Nickel* **OR** Ni **OR** Ni*+ **OR** vehicle* **OR** automobile* **OR** exhaust **OR** motorway* **OR** roadway* **OR** highway* **OR** freeway* **OR** road* **OR** traffic **OR** urban **OR** Nox **OR** "nitrogen oxides" **OR** ozone **OR** particle*)) **OR** dust **OR** dusts **OR** PM*2*5 **OR** PM*10 **OR** "ultrafine particle*" **OR** "polycyclic aromatic hydrocarbon*" **OR** PAH **OR** POPS **OR** smog **OR** "water pollut*" **OR** (water* **AND** (potable **OR** healthy **OR** drink* **OR** safe **OR** suitab* **OR** palatable **OR** edible **OR** tap **OR** fresh **OR** supply **OR** "microbial contamina*")) **OR** waterborne **OR** water*borne **OR** aquifer **OR** groundwater **OR** pesticid* **OR** herbicid* **OR** insecticid* **OR** acaricid* **OR** fungicid* **OR** molluscacid* **OR** larvicid* **OR** fumigant **OR** "anti*fouling agent*" **OR** "agricultural chemical*" **OR** agrochemical* **OR** (defoliant* **AND** (chemical* **OR** agent*)) **OR** (hazardous **AND** substance*) **OR** (toxic **AND** action*) **OR** "chemically*induced disorder*" **OR** furfural **OR** aculeximycin **OR** "aluminum phosphide" **OR** "chromated copper arsenate" **OR** CCA **OR** creosote)
5	3 **OR** 4
6	1 **AND** 2 **AND** 5

References

1. Arksey H, O'Malley L. Scoping studies: towards a methodological framework. Int J Soc Methodol. 2002 Feb;8(1):19–32. DOI: 10.1080/1364557032000119616

2. Levac D, Colquhoun H, O'Brien K. Scoping studies: advancing the methodology. Implement Sci. 2010;5(69). DOI: 10.1186/1748-5908-5-69

3. Aromataris E, Munn Z, editors. Joanna Briggs Institute reviewer's manual. The Joanna Briggs Institute. 2017. Available from https://reviewersmanual.joannabriggs.org/

4. Peters MDJ, Godfrey C, Kahlil H, McInerney P, Baldini Soares C, Parker D. Guidance for conducting systematic scoping reviews. Int J Evid Based Healthc. 2015;13:141–146. DOI: 10.1097/XEB.0000000000000050

5. Moher D, Liberati A, Tetzlaff J, Altman DG. Preferred reporting items for systematic reviews and meta-analyses: the PRISMA statement. Br Med J. 2009 Aug;339(7716):332–336 DOI: 10.1001/jama.2017.19163

6. Tricco AC, Lillie E, Zarin W, O'Brien KK, Colquhoun H, Levac D, et al. PRISMA extension for scoping reviews (PRISMA-ScR): checklist and explanation. Ann Intern Med. 2018 Sept;169:467–473. DOI: 10.7326/M18-0850

7. United Nations Framework Convention on Climate Change. Fact sheet: climate change science - the status of climate change science today. 2011 Feb. Available from https://unfccc.int/files/press/backgrounders/application/pdf/press_factsh_science.pdf

REVISED
8:40 pm, Dec 19, 2020
