# Supplementary material for: One Health, One Hive: A scoping review of honey bees, climate change, pollutants, and antimicrobial resistance
Source: PLoS One. 2022 Feb 16;17(2):e0242393. doi: 10.1371/journal.pone.0242393 (PMC8849492; doi:10.1371/journal.pone.0242393)
Supplement: S2 File — (PDF) [file pone.0242393.s005.pdf]

1. Screening Criteria

Level 1: the following questions will be answered using a stacked form after screening each title and/or abstract:

- Does the research focus on beekeeping and/or honey bee hive health? (altering the normal function or behaviour of the hive or organism)
  - Yes
  - No
  - Unsure
- Does the research focus on AMR? (doesn't include novel drug testing)
  - Yes
  - No
  - Unsure
- Does the research include climatic variables and/or environmental pollutants as points of interest?
  - Yes
  - No
  - Unsure
- Is it a journal article?
  - Yes
  - No
  - Unsure?

If 'unsure' or 'yes' is selected, confirm inclusions.

Level 2: the following questions will be answered after screening each full article:

- Does the research focus on beekeeping and/or honey bee hive health? (altering the normal function or behaviour of the hive or organism)
  - Yes
  - No
- Does the research focus on AMR? (doesn't include novel drug testing)
  - Yes
  - No
- Does the research mention the effects of climatic variables and/or environmental pollutants on AMR development?
  - Yes
  - No

If "yes" is selected for either of the two last questions the article was passed down to data extraction.
