# Supplementary material for: One Health, One Hive: A scoping review of honey bees, climate change, pollutants, and antimicrobial resistance
Source: PLoS One. 2022 Feb 16;17(2):e0242393. doi: 10.1371/journal.pone.0242393 (PMC8849492; doi:10.1371/journal.pone.0242393)
Supplement: S3 File — (PDF) [file pone.0242393.s006.pdf]

Excluded Articles:

1. Austin, U. T. (2013). Megachile rotundata larval guts Targeted Locus (Loci). *European Nucleotide Archive*. Retrieved from http://www.ebi.ac.uk/ena/data/view/PRJNA196833
2. Bassi, S., Milito, M., Giacomelli, A., Carra, E., Cordaro, G., Cersini, A., … Franco, A. (2013). Isolation of P. larvae in bees imported from Argentina: comparison of the “serious” sensitivity to antibiotics of isolates with that of Italian strains [Conference poster]. *XV Congresso Nazionale S.I.Di.L.V., Monreale (PA), Italia, 23-25 Ottobre 2013*, 132–133.
3. Buczek, K. (2009). Honey bee colony collapse disorder (CCD). *Annales Universitatis Mariae Curie-Sklodowska.Sectio DD, Medicina Veterinaria*, *64*(1), 1–6.
4. Chorbinski, P. (2003). Susceptibility of Ascosphaera apis strains to antifungal preparations. *Medycyna Weterynaryjna*, *59*(12), 1137–1139.
5. Drescher, N., Klein, A. M., Neumann, P., Yañez, O., & Leonhardt, S. D. (2017). Inside honeybee hives: Impact of natural propolis on the ectoparasitic mite Varroa destructor and viruses. *Insects*, *8*(1), 15.
6. Dujin, T., Jovanovic, V., & Suvakov, D. (1990). Study of Methods and Therapeuticals Against Varroa-Jacobsoni in Large Hive Colonies Iii. Results of a Four-Year Study of Amitrase-Based Preparations. *Veterinarski Glasnik*, *44*(12), 1107–1115.
7. Dujin, T., Jovanovic, V., Suvakov, D., & Milkovic, Z. (1991). Effects of Extended Utilisation of Amytrase-Based Preparations on the Formation of Resistant Strains of Varroa Varroa-Jacobsoni. *Veterinarski Glasnik*, *45*(11–12), 851–855.
8. Dzierzawski, A., & Cybulski, W. (2010). Evaluation of the efficacy of Apiwarol AS. *Medycyna Weterynaryjna*, *66*(7), 475–479.
9. Faucon, J. P. (1987). Amitraz Treatment of Varroa Disease using Evaporation Or Cold Temperatures. *Sante de l'Abeille*, (98), 65
10. Feldlaufer, M. F., Pettis, J. S., Kochansky, J. P., & Stiles, G. (2001). Lincomycin hydrochloride for the control of American foulbrood disease of honey bees. *Apidologie*, *32*(6), 547-554.
11. Glavinic, U., Tesovnik, T., Stevanovic, J., Zorc, M., Cizelj, I., Stanimirovic, Z., & Narat, M. (2019). Response of adult honey bees treated in larval stage with prochloraz to infection with Nosema ceranae. *PeerJ*, *7*, e6325.
12. Glinski, Z. (1974). [Sensitivity of some strains of Bacillus larvae to antibiotics and

sulphonamides]. *Medycyna Weterynaryjna*, *30*(4), 212–216.

13. Glinski, Z., & Rzedzicki, J. (1977). A Comparison of the Activity of Certain New Sulfonamide Preparations Against Bacillus-Larvae and Other Bacilli Isolated from Honey Bees. *Polskie Archiwum Weterynaryjne*, *20*(3), 9–20.
14. Glinski, Z., & Stark, J. A. (1991). Varroasis Current Research Status and Prospects for its Control. *Medycyna Weterynaryjna*, *47*(1), 10–12.
15. Griselli, B., & Carpana, E. (1997). Monitoring American foul brood by searching Paenibacillus larvae sporae. *Apicoltore Moderno*, *88*(3), 99–105.
16. Guguianu, E., Nicolai, V., Timofte, D., & Carp-Carare, C. (2002). Aspects of culture, identification and resistance to antibiotics of Paenibacillus larvae var. larvae. *Lucrai Stiinifice - Medicina Veterinara, Universitatea de Stiinte Agricole Si Medicina Veterinara "Ion Ionescu de La Brad" Iasi*, *45*(4(2)), 560–564.
17. Hitchcock, J. D. (1966). Bee disease research at Laramie. *Amer Bee J*, *106*((7)), 255.
18. Jevinova, P., Nagy, J., Toporcak, J., & Popelka, P. (2007). Comparison of susceptibility of paenebacillus larvae var. larvae to the antibiotics oxytetracycline and tylosin. *Slovensky Veterinarsky Casopis*, *32*(6), 378–379.
19. Le Conte, Y., Alaux, C., & Vallon, J. (2013). Protecting bees against varroa. *Biofutur*, (343), 48–50.
20. Ludvigsen, J., Porcellato, D., L'Abée-Lund, T. M., Amdam, G. V., & Rudi, K. (2017). Geographically widespread honeybee-gut symbiont subgroups show locally distinct antibiotic-resistant patterns. *Molecular ecology*, *26*(23), 6590-6607.
21. Massimo, C., Michela G. (1986). Sensitivity in vitro of Bacillus larvae strains to various antibiotics and chemotherapeutic agents. *Annali della Facolta di Medicina Veterinaria di Torino*, *31*, 163
22. Mihai, C. M., Mărghitaş, L. A., Dezmirean, D. S., Chirilă, F., Moritz, R. F., & Schlüns, H. (2012). Interactions among flavonoids of propolis affect antibacterial activity against the honeybee pathogen Paenibacillus larvae. *Journal of Invertebrate Pathology*, *110*(1), 68-72.
23. Milkovic, Z., Dujin, T., & Suvakov, D. (1991). Parallel Examinations of Apistan and Fluvalinate Emulsion for the Control of Bee Variosis. *Veterinarski Glasnik*, *45*(11–12), 857–862.

24. Mintiens, K., & Parkin, T. D. H. (2014). *Society for Veterinary Epidemiology and Preventive Medicine, Proceedings of a meeting held in Dublin, Irish Republic, 26-28 March 2014.* (K. P. Mintiens T.D.H., ed.).
25. Raymann, K., Bobay, L. M., & Moran, N. A. (2018). Antibiotics reduce genetic diversity of core species in the honeybee gut microbiome. *Molecular ecology*, *27*(8), 2057-2066.
26. Razavi, S. M., Asadpour, M., Jafari, A., & Malekpour, S. H. (2015). The field efficacy of Lepidium latifolium and Zataria multiflora methanolic extracts against Varroa destructor. *Parasitology research*, *114*(11), 4233-4238.
27. Reid, G. M. (1990). Feeding drugs to honey bees to control diseases - some of the issues. *Feeding Drugs to Honey Bees to Control Diseases - Some of the Issues.*, 26 pp.-26 pp.
28. Rudenko, E. V. (1990). Factors of humoral immunity in bee larvae. *Veterinariya (Kiev)*, *65*, 46–48.
29. Sinanovic, A. L., Mandra, M., Dukic, B., Jasic, M., & Hodzic, S. (2017). Veterinary medicinal products in beekeeping. *Drugi Kongres O Pcelarstvu I Pcelinjim Proizvodima Sa Meddunarodnim Ucescem, 20.Kolovoz 2017, Gradacac, Bosnia i Hercegovina.Zbornik Radova i Sazetaka*, 40.
30. Smolska-Szymczewska, B. (1989). The influence of the chosen chemotherapeutics on the intestinal flora of the honeybee. *Apiacta*, *24*, 71-79.
31. Spivak, M. (2000). Preventative antibiotic treatments for honey bee colonies. *American Bee Journal*, *140*(11), 867-868.
32. Stamets, P. E. (n.d.). *Composition useful for improving bee health and preventing colony collapse disorder among bees comprises nutraceutical product selected from extract of mushroom mycelium, combined with bee product, e.g. sugar syrup, bee candy, sticky strips*.
33. Vandenberg, J. D., Hamm, J. J., & Shimanuki, H. (1986). Vairimorpha sp. spores do not reduce the longevity of caged adult honey bees, Apis mellifera (Hymenoptera: Apidae). *Environmental entomology*, *15*(1), 207-209.
34. Vidal-Naquet, N., & Boucher, S. (2013). The American foulbrood: epidemiology and methods to control it. *Bulletin des GTV*, (68), 101-108.
35. Zoebelein, G., & Kniehase, U. (1985). Laboratory Greenhouse and Field Trials on the Effect of Nikkomycins on Insects and Mites. *Pflanzenschutz-Nachrichten*, *38*(2–3), 203–304.

36. Zubchenkov, V. I., & Smirnova, N. I. (1959). Farm experiment on the sanitation of apiaries from European foulbrood Referat. Zhur., Biol., 1960, No. 30970. (Translation). *Pchelovodstvo*, *8*, 44–48.
